# Supplementary material for: A fault-intrusion-tolerant system and deadline-aware algorithm for scheduling scientific workflow in the cloud
Source: PeerJ Comput Sci. 2021 Nov 2;7:e747. doi: 10.7717/peerj-cs.747 (PMC8576554; doi:10.7717/peerj-cs.747)
Supplement: Supplemental Information 6 [file peerj-cs-07-747-s006.zip › Myproject1.1/docs/org/cloudbus/cloudsim/core/SimEvent.html]

SimEvent


JavaScript is disabled on your browser.


- Overview
- Package
- Class
- Use
- Tree
- Deprecated
- Index
- Help

- Prev Class
- Next Class

- Frames
- No Frames

- All Classes

- Summary:
- Nested |
- Field |
- Constr |
- Method

- Detail:
- Field |
- Constr |
- Method


org.cloudbus.cloudsim.core

## Class SimEvent

- java.lang.Object
- - org.cloudbus.cloudsim.core.SimEvent

- All Implemented Interfaces:
  :   java.lang.Cloneable, java.lang.Comparable<SimEvent>

  ---

    

  ```
  public class SimEvent
  extends java.lang.Object
  implements java.lang.Cloneable, java.lang.Comparable<SimEvent>
  ```

  This class represents a simulation event which is passed between the entities in the simulation.

  See Also:
  :   `Simulation`,
      `SimEntity`

- - ### Field Summary

    Fields

    | Modifier and Type | Field and Description |
    | --- | --- |
    | `static int` | `CREATE` |
    | `static int` | `ENULL` |
    | `static int` | `HOLD_DONE` |
    | `static int` | `SEND` |
  - ### Constructor Summary

    Constructors

    Constructor and Description

    `SimEvent()`

    Create a blank event.
  - ### Method Summary

    Methods

    | Modifier and Type | Method and Description |
    | --- | --- |
    | `java.lang.Object` | `clone()` Create an exact copy of this event. |
    | `int` | `compareTo(SimEvent event)` |
    | `double` | `endWaitingTime()` Get the simulation time that this event was removed from the queue for service. |
    | `double` | `eventTime()` Get the simulation time that this event was scheduled. |
    | `java.lang.Object` | `getData()` Get the data passed in this event. |
    | `int` | `getDestination()` Get the unique id number of the entity which received this event. |
    | `int` | `getSource()` Get the unique id number of the entity which scheduled this event. |
    | `int` | `getTag()` Get the user-defined tag of this event. |
    | `int` | `getType()` The internal type |
    | `int` | `scheduledBy()` Get the unique id number of the entity which scheduled this event. |
    | `void` | `setDestination(int d)` Set the destination entity of this event. |
    | `protected void` | `setEndWaitingTime(double end_waiting_time)` Used to set the time at which this event finished waiting in the event |
    | `protected void` | `setSerial(long serial)` |
    | `void` | `setSource(int s)` Set the source entity of this event. |
    | `java.lang.String` | `toString()` |
    | `int` | `type()` Get the user-defined tag of this event |

    - ### Methods inherited from class java.lang.Object

      `equals, finalize, getClass, hashCode, notify, notifyAll, wait, wait, wait`

- - ### Field Detail


    - #### ENULL

      ```
      public static final int ENULL
      ```

      See Also:
      :   Constant Field Values


    - #### SEND

      ```
      public static final int SEND
      ```

      See Also:
      :   Constant Field Values


    - #### HOLD\_DONE

      ```
      public static final int HOLD_DONE
      ```

      See Also:
      :   Constant Field Values


    - #### CREATE

      ```
      public static final int CREATE
      ```

      See Also:
      :   Constant Field Values
  - ### Constructor Detail


    - #### SimEvent

      ```
      public SimEvent()
      ```

      Create a blank event.
  - ### Method Detail


    - #### setSerial

      ```
      protected void setSerial(long serial)
      ```


    - #### setEndWaitingTime

      ```
      protected void setEndWaitingTime(double end_waiting_time)
      ```

      Used to set the time at which this event finished waiting in the event

      Parameters:
      :   `end_waiting_time` -


    - #### toString

      ```
      public java.lang.String toString()
      ```

      **Overrides:**
      :   `toString` in class `java.lang.Object`


    - #### getType

      ```
      public int getType()
      ```

      The internal type

      Returns:


    - #### compareTo

      ```
      public int compareTo(SimEvent event)
      ```

      **Specified by:**
      :   `compareTo` in interface `java.lang.Comparable<SimEvent>`

      See Also:
      :   `Comparable.compareTo(Object)`


    - #### getDestination

      ```
      public int getDestination()
      ```

      Get the unique id number of the entity which received this event.

      Returns:
      :   the id number


    - #### getSource

      ```
      public int getSource()
      ```

      Get the unique id number of the entity which scheduled this event.

      Returns:
      :   the id number


    - #### eventTime

      ```
      public double eventTime()
      ```

      Get the simulation time that this event was scheduled.

      Returns:
      :   The simulation time


    - #### endWaitingTime

      ```
      public double endWaitingTime()
      ```

      Get the simulation time that this event was removed from the queue for service.

      Returns:
      :   The simulation time


    - #### type

      ```
      public int type()
      ```

      Get the user-defined tag of this event

      Returns:
      :   The tag


    - #### scheduledBy

      ```
      public int scheduledBy()
      ```

      Get the unique id number of the entity which scheduled this event.

      Returns:
      :   the id number


    - #### getTag

      ```
      public int getTag()
      ```

      Get the user-defined tag of this event.

      Returns:
      :   The tag


    - #### getData

      ```
      public java.lang.Object getData()
      ```

      Get the data passed in this event.

      Returns:
      :   A reference to the data


    - #### clone

      ```
      public java.lang.Object clone()
      ```

      Create an exact copy of this event.

      **Overrides:**
      :   `clone` in class `java.lang.Object`

      Returns:
      :   The event's copy


    - #### setSource

      ```
      public void setSource(int s)
      ```

      Set the source entity of this event.

      Parameters:
      :   `s` - The unique id number of the entity


    - #### setDestination

      ```
      public void setDestination(int d)
      ```

      Set the destination entity of this event.

      Parameters:
      :   `d` - The unique id number of the entity


- Overview
- Package
- Class
- Use
- Tree
- Deprecated
- Index
- Help

- Prev Class
- Next Class

- Frames
- No Frames

- All Classes

- Summary:
- Nested |
- Field |
- Constr |
- Method

- Detail:
- Field |
- Constr |
- Method
